# Does Climate Warming Favour Early Season Species?

**DOI:** 10.3389/fpls.2021.765351

**Published:** 2021-11-18

**Authors:** Xiuli Chu, Rongzhou Man, Haicheng Zhang, Wenping Yuan, Jing Tao, Qing-Lai Dang

**Affiliations:** ^1^Shanghai Botanical Garden, Shanghai Engineering Research Center of Sustainable Plant Innovation, Shanghai, China; ^2^Ontario Ministry of Northern Development, Mines, Natural Resources and Forestry, Ontario Forest Research Institute, Sault Ste. Marie, ON, Canada; ^3^Department Geoscience, Environment and Society, Université Libre de Bruxelles, Brussels, Belgium; ^4^School of Atmospheric Science, Sun Yat-sen University, Guangzhou, China; ^5^Jilin Provincial Academy of Forestry Sciences, Changchun, China; ^6^Faculty of Natural Resources Management, Lakehead University, Thunder Bay, ON, Canada

**Keywords:** leaf-out, growing season length, forcing gains, chilling effects, interspecific differences, phenological responses, leaf-out temperature

## Abstract

Plant species that start early in spring are generally more responsive to rising temperatures, raising concerns that climate warming may favour early season species and result in altered interspecific interactions and community structure and composition. This hypothesis is based on changes in spring phenology and therefore active growing season length, which would not be indicative of possible changes in growth as would changes in cumulative forcing temperatures (growing degree days/hours) in the Northern Hemisphere. In this study we analysed the effects of a moderate climate warming (2°C warmer than the 1981–2010 baseline) on the leaf-out of hypothetical species without chilling restriction and actual plant species with different chilling and forcing requirements in different parts of the globe. In both cases, early season species had larger phenological shifts due to low leaf-out temperatures, but accumulated fewer forcing gains (changes in cumulative forcing temperatures by warming) from those shifts because of their early spring phenology. Leaf-out time was closely associated with leaf-out temperatures and therefore plant phenological responses to climate warming. All plant species would be equally affected by climate warming in terms of total forcing gains added from higher temperatures when forcing gains occurring between early and late season species are included. Our findings will improve the understanding of possible mechanisms and consequences of differential responses in plant phenology to climate warming.

## Introduction

Plant phenology, particularly in spring, is sensitive to temperature changes (Murray et al., 1989; Wang et al., 2015; Primack and Gallinat, 2016) and therefore responsive to climate warming (Badeck et al., 2004; Schwartz et al., 2006; Gunderson et al., 2012). Extensive data have been accumulated through observational studies and warming experiments (Parmesan, 2007; Wolkovich et al., 2012). Interpreting phenological changes and understanding differences among species in their responses to climate warming, however, are challenging. In addition to variations across geographic gradients of latitudes and altitudes (Root et al., 2003; Ziello et al., 2009; Čufar et al., 2012; Prevéy et al., 2017; Post et al., 2018; Vitasse et al., 2018; Suonan et al., 2019), plant phenological responses vary substantially among species with differing spring phenology, i.e., species that leaf-out early in spring are generally more responsive to changes in temperatures than those that leaf-out later (Morin et al., 2009; Polgar et al., 2014; Shen et al., 2014; Primack and Gallinat, 2016), resulting in increasing interspecific differences in leaf duration and potentially in seasonal photosynthesis and growth (Badeck et al., 2004; Vitasse et al., 2009; Polgar and Primack, 2011; Cleland et al., 2012; Gunderson et al., 2012). If larger phenological advances enable early season plants to track temperature changes and exploit an earlier spring, as is often suggested (Fitter and Fitter, 2002; Primack and Gallinat, 2016; Zettlemoyer et al., 2019), these early plants could gain growth and competitive advantages over late season species. It is therefore widely believed that climate warming favours early season species and could alter interspecific relationships, community structure, and ecosystem functions (Fitter and Fitter, 2002; Polgar and Primack, 2011; Polgar et al., 2014; Shen et al., 2014; Primack and Gallinat, 2016).

The implications of interspecific differences in phenological responses, however, would be different if plant responses to climate warming are assessed by changes in cumulative forcing temperatures that occur during phenological changes. During the spring, daily forcing temperatures change substantially over time, as air temperatures increase. On the one hand, early season species have more changes in spring phenology per unit forcing temperature due to low leaf-out temperatures and are therefore more sensitive to temperature changes (Fitter and Fitter, 2002; Shen et al., 2014; Zhang et al., 2015). On the other hand, early season species would have smaller changes in cumulative forcing temperatures from warming than late season species due to their early spring phenology and cumulative effects of spring temperatures. It is not clear how changes in spring phenology are associated with changes in cumulative forcing temperatures among species with differing spring phenology. Past plant phenological research are generally focused on changes in spring phenology, although changes in cumulative forcing temperatures generally drive plant development in the Northern Hemisphere (Hänninen, 1990; Badeck et al., 2004; Man and Lu, 2010).

In this study, we assessed plant responses to warming by changes in both leaf-out time (phenological shifts/changes/responses) and cumulative forcing temperatures (growing degree days/hours). This assessment was based on plant responses under different scenarios of chilling satisfaction, hypothetical species without chilling restriction and actual plant species with different chilling and forcing requirements (species-specific leaf-out models developed in Canada, China, and United Kingdom). Our objective was to determine how spring phenology may influence plant phenological shifts and if phenological shifts would represent possible changes in plant growth due to climate warming. More specifically, we were interested in knowing (1) if leaf-out time and forcing requirements (species-specific cumulative forcing temperatures for leaf-out) are associated with insufficient chilling induced by warming climate and (2) how leaf-out time, leaf-out temperature, phenological shifts, and forcing gains (changes in cumulative forcing temperatures by warming) relate to each other.

## Materials and Methods

**FIGURE 1:**
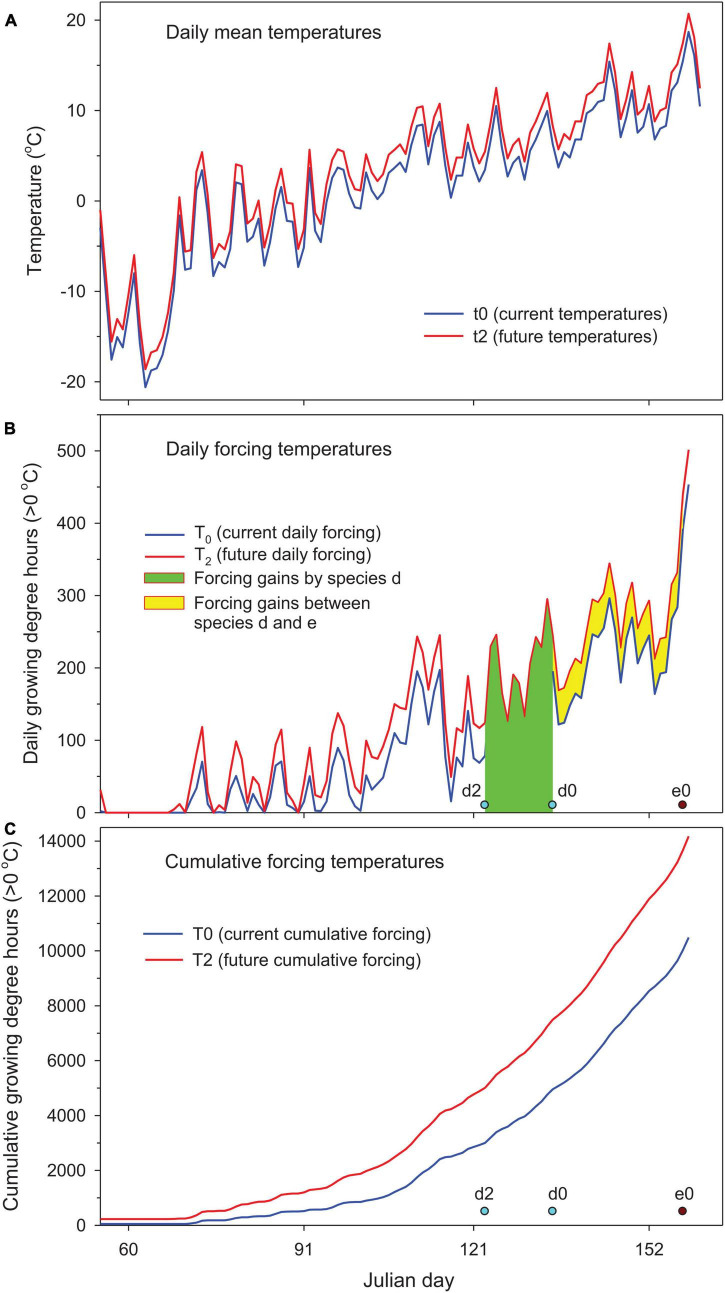
Quantification of species response to climate warming (2°C uniform warming) for hypothetical species d (forcing requirement = 5,000 cumulative growing degree hours (CGDH > 0°C) since January 1 at leaf-out date d0 and d2 under current and future warmer conditions) relative to reference species e (10,000 CGDH at e0) using 2018–2019 winter temperatures in Sault Ste Marie, Ontario, Canada, **(A)** mean daily temperatures, **(B)** daily forcing temperatures showing forcing gains by species d (Equation 4 in “Materials and Methods”) and forcing gains that occur between subject species d and reference species e (Equation 5 in “Materials and Methods”), and **(C)** cumulative forcing temperatures (see Table 1 for more details).

To illustrate how plants with differing spring phenology would respond to warmer (+2°C) temperatures, we first examined hypothetical species with different forcing requirements using temperature data from Sault Ste. Marie, Ontario, Canada (Figure 1 and Table 1). Chilling requirements for breaking winter dormancy were assumed to be fully met before the start of forcing temperature accumulation from January 1 to ensure that phenological responses were not restricted by possible insufficient chilling induced by warming. This assumption generally holds for this area because of the long, cold winters with temperatures below zero until early March (Figure 1A; Colombo, 1998; Man et al., 2017, 2020). We chose five levels of forcing requirements for leaf-out: 500, 1,000, 2,000, 5,000, and 10,000 growing degree hours >0°C since January 1. The first two represent very early spring plants such as grasses and early shrubs (Badeck et al., 2004; Primack and Gallinat, 2016), while the other three represent tree species with early to late spring phenology (Colombo, 1998; Man et al., 2017, 2020).

**TABLE 1:** Phenological shifts (changes in leaf-out time), forcing gains (changes in cumulative forcing temperatures), and leaf-out temperatures (average temperatures within the window of phenological shifts) for hypothetical species of very early to late spring phenology [500–10,000 cumulative growing degree hours (CGDH > 0°C) since January 1] in response to 2°C uniform increases of 2018–2019 winter temperatures in Sault Ste Marie, Ontario, Canada.

Species	a	b	c	d	e
Forcing requirement (CGDH)	500	1,000	2,000	5,000	10,000
Phenological shift (days)	15.3	18.6	10.4	12.6	12.6
Forcing gains from phenological shift (CGDH)	647	1,184	1,540	2,558	3,636
Chilling effects (CGDH)	0	0	0	0	0
Forcing gains by species (CGDH)	647	1,184	1,540	2,558	3,636
Forcing gains between species (CGDH)	2,989	2,452	2,096	1,078	Reference
Total forcing gains in spring (CGDH)	3,636	3,636	3,636	3,636	3,636
Leaf-out temperature (°C)	1.76	2.65	6.16	8.49	12.02

*To evaluate species responses to warming without chilling restriction (chilling effects = 0), chilling requirements are assumed to be fully met prior to accumulation of spring forcing temperatures by January 1 (see “Materials and Methods”).*

We then compared the responses of woody plant species with differing spring phenology in response to 2°C uniform increase of hourly or daily temperatures over the 1981–2010 baseline, using species-specific chilling–forcing models developed under different climates. Both phenological shifts and forcing gains were averaged over 30 years and 9–12 locations for each species (Supplementary Table 1). Species phenological shifts were related to their forcing requirements and leaf-out temperatures using linear or nonlinear regression models for each of the five regions—boreal Canada, temperate Canada, cold temperate China, warm temperate China, and temperate United Kingdom—using R version 3.6.2 (R Development Core Team, 2019).

### Phenological Models and Predictions

We selected leaf-out models developed in different climate conditions for species with differing spring phenology (Supplementary Table 2). Three studies met our needs, 3-parameter exponential and 4-parameter sigmoid models for boreal and temperate trees in Canada (Man et al., 2017, 2020), 2-parameter exponential models for temperate trees/shrubs in China (Zhang et al., 2018), and 3-parameter exponential models for temperate trees/shrubs in the United Kingdom (Murray et al., 1989). The hourly temperatures required by leaf-out models for boreal and temperate tree species in Canada (Man et al., 2017, 2020) were downloaded from the Environment Canada historical data portal^1^ for 12 locations in the boreal region and 9 locations in the temperate region across the country (Supplementary Table 1). Daily temperature data for the species in China were obtained from the China Meteorological Data Sharing Service System^2^ for 24 weather stations (12 each for cold and warm temperate regions). For the United Kingdom, temperature data were obtained from the Met Office gridded land surface climate observations (Hollis and McCarthy, 2017) for 5 km × 5 km grids covering 12 locations (Supplementary Table 1).

We followed the original researchers’ methods in calculation of winter chilling and forcing temperature (growing degree days/hours). The hourly chilling required for the leaf-out models in Canada was calculated using the Sarvas model (Sarvas, 1974) and accumulated from October 1 of the previous year and cumulative forcing temperatures were calculated as cumulative growing degree hours above 0°C starting from January 1 of the current year (Man et al., 2017, 2020). For species in China, winter chilling and forcing temperatures were accumulated from November 1 and January 1, respectively, using daily mean temperatures and species-specific threshold parameters (a phenology model referred to as Ar3 in the original study) (Zhang et al., 2018). For species in the United Kingdom, winter chilling was determined as the number of days (mean daily temperature <5°C since November 1, previous year) and cumulative forcing temperatures as cumulative growing degree days (mean daily temperature >5°C since January 1, current year) (Murray et al., 1989). For all species and locations, leaf-out time was determined when the chilling–forcing relationships of the species/species groups crossed the chilling–forcing curves of weather data. Because of differences in units (day or hour) and thresholds in forcing temperature calculations, we used cumulative mean daily temperatures (>5°C since January 1) at the estimated leaf-out time for comparing forcing gains and forcing requirements among species.

### Quantifying Species Responses to Warming

For both hypothetical species and actual woody species with differing chilling and forcing requirements, their responses to warmer temperatures were assessed by phenological shifts and forcing gains. Unlike phenological shift that only measures changes in leaf-out time, the forcing gains by individual species need to be partitioned between shifting spring phenology and balancing chilling effects due to warming-induced variations in species’ forcing requirements. The forcing gains between phenological events of different species should also be considered when species with differing spring phenology are compared with each other. The forcing gains by different reference points can be quantified, as outlined below, from cumulative forcing (cumulative forcing temperatures since January 1) or daily forcing temperatures (illustrated for species d, see Figure 1).


(1) P⁢h⁢e⁢n⁢o⁢l⁢o⁢g⁢i⁢c⁢a⁢l⁢s⁢h⁢i⁢f⁢t=a⁢b⁢s⁢|d⁢0-d⁢2|

where d0 and d2 are the current (2018–2019 baseline) and future (2°C warmer) dates of spring phenology (leaf-out, see Figure 1B). Spring phenology could advance, i.e., d2 < d0, or be delayed due to insufficient chilling, i.e., d2 > d0. Absolute values were used to conserve the variations of phenological shifts in the calculation of averages over multiple years and locations.


(2) F⁢o⁢r⁢c⁢i⁢n⁢g⁢g⁢a⁢i⁢n⁢s⁢f⁢r⁢o⁢m⁢p⁢h⁢e⁢n⁢o⁢l⁢o⁢g⁢i⁢c⁢a⁢l⁢s⁢h⁢i⁢f⁢t={∑d⁢0d⁢2T2⁢i,on⁢daily⁢forcing⁢(Figure⁢ 1⁢B) T⁢2⁢(d⁢0)-T⁢2⁢(d⁢2),on⁢cumulative⁢forcing⁢(Figure⁢ 1⁢C) T⁢2⁢(d⁢0)-T⁢0⁢(d⁢0),T2⁢(d2)=T0⁢(d0)⁢(Figure⁢ 1⁢C) 

The forcing gains that occur during phenological shifts can be quantified from daily forcing temperatures or cumulative forcing temperatures. Here, T_2i_ is the future (2°C warmer) daily forcing temperatures (Figure 1B), and T0 and T2 are the current and future cumulative forcing temperatures (Figure 1C). In a simplified scenario (option 3) where forcing requirements do not change with warming, the forcing gains from phenological shifts can be calculated from the differences between current and future cumulative forcing temperatures by the current date of spring event.


(3) Chillingeffects=T2(d2)--T0(d0)

The differences in species’ forcing requirement between current (T0(d0)) and future warmer (T2(d2)) conditions can be attributed to chilling effects when winter chilling is inadequate and varies with warming. Chilling effects are positive when forcing requirement increases or negative when forcing requirement decreases with warming.


(4) $$F\,o\,r\,c\,i\,n\,g\,g\,a\,i\,n\,s\,b\,y\,s\,p\,e\,c\,i\,e\,s=\left\{ \begin{array}{c} f\,o\,r\,c\,i\,n\,g\,g\,a\,i\,n\,s\,f\,r\,o\,m\,p\,h\,e\,n\,o\,l\,o\,g\,i\,c\,a\,l\,s\,h\,i\,f\,t+c\,h\,i\,l\,l\,i\,n\,g\,e\,f\,f\,e\,c\,t\,s\\ T\,2\,\left(d\,0\right)-T\,0\,\left(d\,0\right),\mathrm{on}\,\mathrm{cumulative}\,\mathrm{forcing} \end{array}\right.$$

The forcing gains by species can be determined with forcing gains from phenological shifts and chilling effects, or calculated directly from cumulative forcing temperatures. Species phenological shifts would be less than expected from warming when chilling effects > 0, i.e., forcing gains from phenological shifts < forcing gains by species, or more than expected from warming when chilling effects < 0, i.e., forcing gains from phenological shifts > forcing gains by species.


(5) $$F\,o\,r\,c\,i\,n\,g\,g\,a\,i\,n\,s\,b\,e\,t\,w\,e\,e\,n\,s\,p\,e\,c\,i\,e\,s=\left\{ \begin{array}{c} \left[T\,2\,\left(e\,0\right)-T\,0\,\left(e\,0\right)\right]-\left[T\,2\,\left(d\,0\right)-T\,0\,\left(d\,0\right)\right],\\ \mathrm{on}\,\mathrm{cumulative}\,\mathrm{forcing}\\ \sum_{d\,0}^{e\,0}T_{2\,i}-\sum_{d\,0}^{e\,0}T_{0\,i},\\ \mathrm{on}\,\mathrm{daily}\,\mathrm{forcing} \end{array}\right.$$

The forcing gains between species are the forcing gains occurring in one species relative to that of another (i.e., species d relative to reference species e, or alternatively to a date in late spring when all plants start growing, e.g., May 31) and equal the differences between forcing gains by reference species e and those by subject species d. Here e0 is the current date of spring phenology for reference species e. The forcing gains beyond reference species do not differ among species being compared and therefore do not need to be considered.


(6) Total forcing gains = forcing gains by species + forcing gains between species

The total forcing gains in spring are the total forcing gains for the species being compared and increase with climate warming and spring phenology for the reference species.


(7) $$L\,e\,a\,f\,\text{-}\,o\,u\,t\,t\,e\,m\,p\,e\,r\,a\,t\,u\,r\,e=\sum_{d\,0}^{d\,2}t\,2/P\,h\,e\,n\,o\,l\,o\,g\,i\,c\,a\,l\,s\,h\,i\,f\,t$$

Leaf-out temperature is the average temperature within the time window of the phenological shift (d0 to d2) on future daily mean temperatures (t2) (Figure 1A).

## Results

### Hypothetical Species Without Chilling Restriction

The hypothetical species that have lower forcing requirements (early season species) generally had larger phenological shifts but fewer forcing gains from phenological shifts (equivalent to forcing gains by species due to absence of chilling effects), compared to the species that have higher forcing requirements (late season species) (Table 1). While the forcing gains by species increased with leaf-out time (i.e., species’ forcing requirement), the association of phenological shifts with leaf-out time was strongly influenced by temperature fluctuations during the period of phenological shifts, i.e., smaller shifts with relatively high leaf-out temperatures (more concentrated forcing temperatures, species c and d) and larger shifts with relatively low leaf-out temperatures (more spread forcing temperatures, species b). Total forcing gains in spring were the same for all species after forcing gains occurring between species (relative to late season species e) were added to forcing gains by species (Figure 1 and Table 1).

### Woody Species With Differing Chilling and Forcing Requirements

**FIGURE 2:**
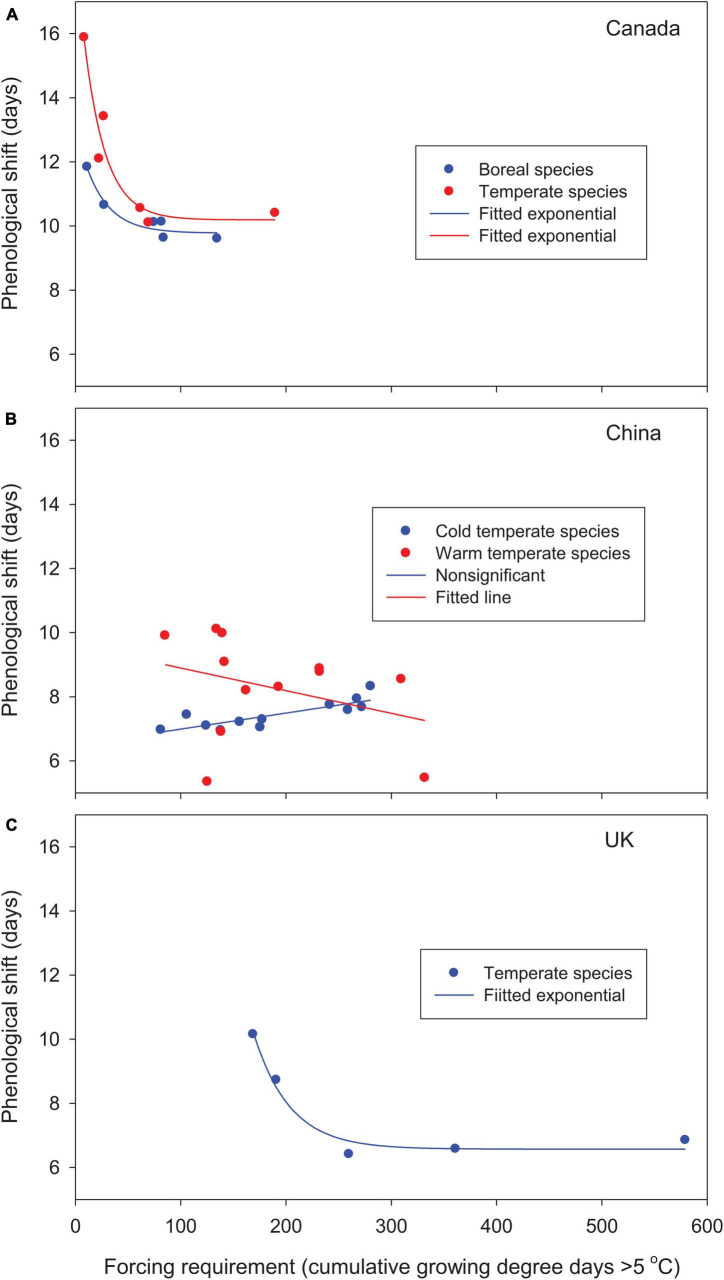
Relationships between phenological shifts of woody species from the 1981–2010 baseline to future warmer (2°C uniform warming) climates and species’ forcing requirements (cumulative growing degree days > 5°C since January 1 at leaf-out) for **(A)** Canada, **(B)** China, and **(C)** the United Kingdom. Values are averaged by species/species groups over 30 years and 9–12 locations under baseline and future warmer climates. Species forcing requirements range from low to high for early to late season species.

**FIGURE 3:**
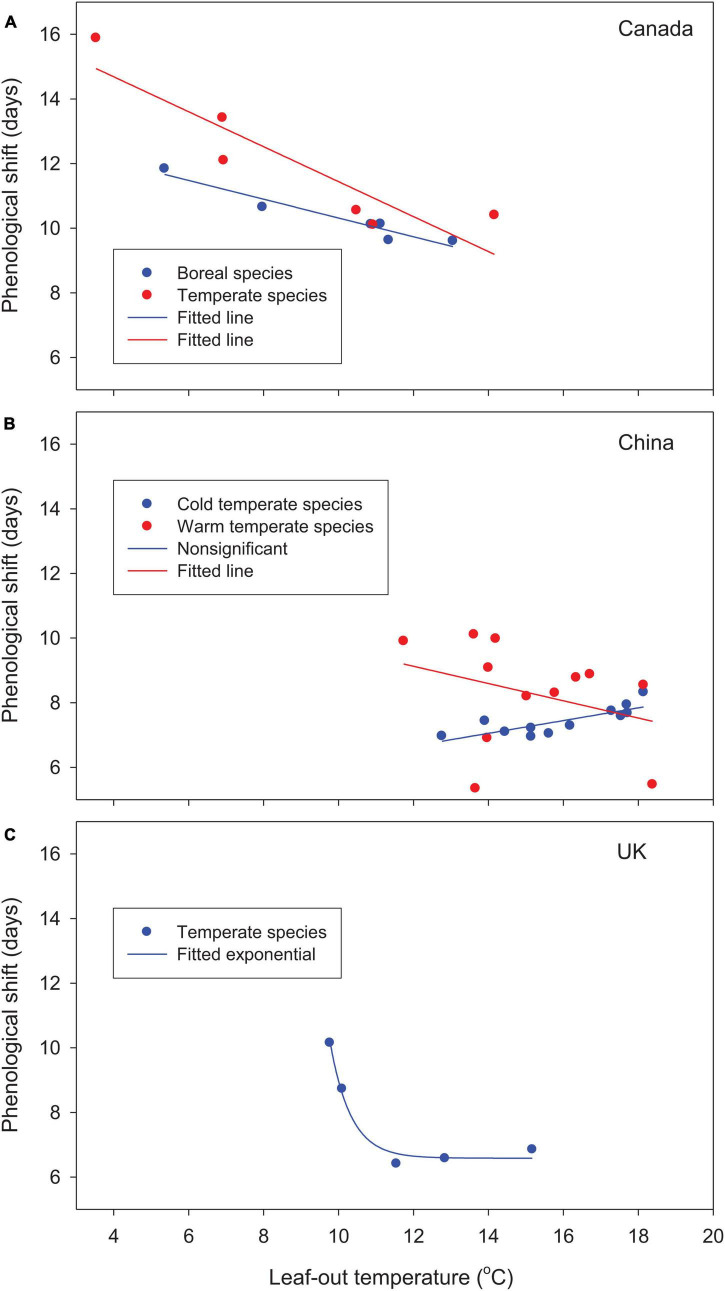
Relationships between phenological shifts of woody species from the 1981–2010 baseline to future warmer (2°C uniform warming) climates and leaf-out temperatures (average temperatures during phenological shifts) for **(A)** Canada, **(B)** China, and **(C)** the United Kingdom. Values are averaged by species/species groups over 30 years and 9–12 locations under baseline and future warmer climates.

For the woody species selected from different parts of the globe, leaf-out time, averaged over multiple years and locations, advanced by 10–16 days in Canada and 5–10 days in China relative to the 1981–2010 baseline. In the United Kingdom, leaf phenology was either advanced or delayed (positive or negative forcing gains from phenological shift; see Table 2). Except for cold temperate species in China, based on best-fit regression models, phenological shifts decreased significantly with increases in species’ forcing requirements from early to late spring phenology and leaf-out temperatures (Figures 2, 3). Early season species generally had larger phenological shifts than late season species. These trends were more evident in Canada and the United Kingdom, where the study species have broader ranges of spring phenology (Figure 2) and more variable leaf-out temperatures during phenological shifts (Figure 3).

**TABLE 2:** Mean forcing gains (changes in cumulative growing degree days > 5°C since January 1) from 2°C uniform increases of 1981–2010 temperatures by species/species groups and climate regions (averages over 30 years and 9–12 locations^a^).

Species/species group	Phenological shift (1)	Chilling effect (2)	By species (1 + 2)	Between species (3)	Total in spring (1 + 2 + 3)
**Boreal species in Canada (12 locations)**					
*Pinus banksiana*	+16	−1	+15	+53	68
*Populus balsamifera*	+30	−3	+27	+41	68
*Betula papyrifera*	+52	−4	+48	+20	68
*Populus tremuloides*	+55	−4	+51	+17	68
*Picea glauca*	+54	−2	+52	+16	68
*Picea mariana*	+70	−2	+68	Reference	68
**Temperate species in Canada (9 locations)**					
*Pinus strobus*	+13	−1	+12	+72	84
*Larix laricina*	+29	−5	+24	+60	84
*Betula alleghaniensis*	+37	−11	+26	+58	84
*Pinus resinosa*	+52	−9	+43	+41	84
*Thuja occidentalis*	+54	−8	+46	+38	84
*Picea rubens*	+91	−7	+84	Reference	84
**Cold temperate species in China (12 locations)**					
*Salix babylonica*	+48	−2	+46	+46	92
*Salix matsudana*	+58	−5	+53	+39	92
*Populus simonii*	+60	−2	+58	+34	92
*Ulmus pumila*	+63	−1	+62	+30	92
*Populus X canadensis*	+70	−4	+66	+26	92
*Amorpha fruticosa*	+71	−1	+70	+22	92
*Koelreuteria paniculata*	+75	−4	+71	+21	92
*Fraxinus chinensis*	+89	−4	+85	+7	92
*Ailanthus altissima*	+91	−3	+88	+4	92
*Ginkgo biloba*	+96	−7	+89	+3	92
*Morus alba*	+94	−4	+90	+2	92
*Sophora japonica*	+102	−10	+92	Reference	92
**Warm temperate species in China (12 locations)**					
*Prunus davidiana*	+60	−3	+57	+51	108
*Platycladus orientalis*	+43	+23	+66	+42	108
*Juglans regia*	+80	−8	+72	+36	108
*Malus pumila*	+58	+13	+71	+37	108
*Euonymus alatus*	+81	−7	+74	+34	108
*Prunus kansuensis*	+76	−2	+74	+34	108
*Amygdalus persica*	+77	+1	+78	+30	108
*Robinia pseudoacacia*	+85	+1	+86	+22	108
*Gleditsia sinensis*	+98	−4	+94	+14	108
*Toona sinensis*	+96	−2	+94	+14	108
*Ziziphus jujuba*	+108	0	+108	Reference	108
*Pinus tabuliformis*	+71	+38	+109	−1	108
**Temperate species in the United Kingdom (12 locations)**					
Group 1*^b^*	−8	+122	+114	+132	246
Group 2	+32	+104	+136	+110	246
Group 3	+24	+139	+163	+83	246
Group 4	+40	+161	+201	+45	246
Group 5	−30	+276	+246	Reference	246

*^a^See Supplementary Table 1 for detailed location information. Forcing gains by species go to phenological shifts (± for advanced/delayed leaf-out time) and chilling effects due to warming-induced variations in forcing requirements (± for increased/decreased forcing requirements due to insufficient chilling). Forcing gains between species are the forcing gains occurring between subject and reference species and can be positive or negative depending on phenological differences between species. Total forcing gains in spring (>0) represent changes in cumulative forcing temperatures due to warming for the compared species. Within each climate region, species are listed by increasing forcing requirements from early to late spring phenology.*

*^b^See Supplementary Table 2 for species included in each group.*

The cold temperate species in China showed slightly different, but non-significant trends. The initiation of leaf-out occurred in late spring at higher leaf-out temperatures, which reduced phenological shifts, particularly for early season species, and resulted in smaller interspecific differences. The large increase in species’ forcing requirements in the warm temperate species *Platycladus orientalis* and *Pinus tabuliformis* by warming (chilling effect; see Table 2) reduced phenological shifts (2 outliers in Figures 2, 3).

Warming increased winter chilling and therefore reduced species’ forcing requirements for Canadian species and cold temperate species in China, as shown by negative chilling effects for all species (Table 2). The mixed changes in species’ forcing requirements, as indicated by both positive and negative chilling effects, among warm temperate species in China resulted in variable phenological shifts, whereas the large increases in species’ forcing requirements among species in the United Kingdom (high positive chilling effects) substantially reduced the allocation of species forcing gains on phenological shifts (Figures 2, 3 and Table 2).

## Discussion

The smaller phenological shifts for late season species are often attributed to their higher chilling requirements and therefore insufficient chilling induced by warming climate (Morin et al., 2009; Polgar et al., 2014; Primack and Gallinat, 2016). Our analyses do not support such a connection. Late season species were less responsive to warming, even without chilling restriction (hypothetical species with zero chilling effects). For woody species examined in this study, insufficient chilling (positive chilling effects) occurred only in species in warm temperate China and the United Kingdom and was associated with species forcing requirements only for United Kingdom species (Table 2). When chilling effects were expressed in days of phenological changes, however, no such relationship was evident. This result is consistent with previous findings by some that species chilling requirements are not associated with their timing of spring events (Man et al., 2017, 2020), but contrary to the suggestions by many others that they are (Morin et al., 2009; Polgar et al., 2014; Primack and Gallinat, 2016).

Our leaf-out temperature and forcing gain analyses provide an innovative approach to understanding plant phenological responses to climate warming. Our findings help explain varying phenological sensitivity among species that has long been noticed but not well understood, i.e., early season species and vegetation are more sensitive to temperature changes and more responsive to climate warming (Fitter and Fitter, 2002; Shen et al., 2014; Zhang et al., 2015). The primary difference between early and late season species is the forcing requirement to initiate spring events, but not necessarily the need for winter chilling to release dormancy. Although the forcing gains by species are smaller for early season species, they are subject to lower leaf-out temperatures in early spring and therefore require larger phenological shifts to accumulate forcing temperatures. Comparatively, late season species can accumulate more forcing temperatures in less time because temperatures are higher. The slow accumulation of forcing temperatures under low temperatures at the time of spring events would also help explain the larger phenological responses at high latitudes and altitudes (Root et al., 2003; Ziello et al., 2009; Čufar et al., 2012; Prevéy et al., 2017; Post et al., 2018; Vitasse et al., 2018; Suonan et al., 2019) and for earlier phenological events (Ma et al., 2021). Therefore, differences in temperatures and rates of forcing temperature accumulations at the time of spring events may largely account for interspecific and regional differences in temperature sensitivity and phenological responses.

Our analyses indicate that interspecific differences in spring phenology and active growing season length will increase with climate warming (Badeck et al., 2004; Vitasse et al., 2009; Gunderson et al., 2012). This increasing spread of timing and duration of active growing season, however, would not alter interspecific relationships, community structure, or ecosystem function. Our results showed that phenological responses do not represent species forcing gains, that chilling effects are not associated with spring phenology, and that all species are equally affected by warming in terms of total forcing gains in spring from higher temperatures. The use of phenological responses or active growing season extensions by changes in spring phenology in modelling and projection, as is often suggested (Peñuelas et al., 2009; Cleland et al., 2012; Prevéy et al., 2017), would include more early spring days of low temperatures and therefore overestimate the extra growth that may occur due to warming for early season species. By contrast, the use of forcing gains by species would miss between species gains and lead to underestimation for early season species. The larger phenological responses by early season species do not bring thermal advantages, contrary to some common beliefs (Fitter and Fitter, 2002; Polgar and Primack, 2011; Polgar et al., 2014; Shen et al., 2014; Primack and Gallinat, 2016) that are apparently based on the assumption that early spring cold days contribute equally to plant growth as would late spring warm days. This assumption overlooks the fact that in the Northern Hemisphere, spring temperatures are generally below the optimum for plant growth; the earlier the spring phenology, the lower the leaf-out temperature and therefore the more plant growth is likely to be restricted (Tao et al., 2021). In boreal Canada, some early season species start leaf-out at daily temperatures slightly above zero (Figures 1, 3 and Table 1). New leaf development would be slow or stagnant in early and cold springs (Polgar and Primack, 2011). Although evergreen mature leaves can resume photosynthesis as soon as temperatures reach above zero, their photosynthetic systems would not operate at high efficiency (Man and Lieffers, 1997a), particularly with cold soils (Man and Lieffers, 1997b). Exposure to high irradiance at low photosynthetic capacity in late winter and early spring can lead to the destruction of photosynthetic systems through photoinhibition and photooxidation (Man and Lieffers, 1997b).

The larger phenological responses by early season species and more species forcing gains by late season species are both true from individual species perspective but biased and misleading for among-species comparisons when interspecific differences in spring phenology and chilling effects are not considered. Among the four species response attributes examined (i.e., phenological shifts, forcing gains from phenological shifts, forcing gains by species, and chilling effects), chilling effects are the only one that is not associated with leaf-out temperatures or timing of spring events and can help detect among-species differences in potential growth responses to climate warming. Species with high positive chilling effects would have disadvantages, whereas those with more negative chilling effects would have advantages over other species. As early successional and invasive species generally start growing early in spring (Körner and Basler, 2010; Polgar and Primack, 2011; Polgar et al., 2014), their sensitive spring phenology and larger increases in active growing season length result in competitive advantages and therefore potential changes in ecosystem structure and function under future climate (Fitter and Fitter, 2002; Morin et al., 2009; Polgar and Primack, 2011; Polgar et al., 2014; Shen et al., 2014; Primack and Gallinat, 2016; Zettlemoyer et al., 2019). In forestry, the conifer-based wood industry would change due to competitive disadvantages and therefore possible decline of late season conifers on the landscape. Based on our forcing gain analyses, this would not occur. Instead, early season species may have disadvantages if larger advances in spring phenology are associated with higher risk of frosts (Morin et al., 2009; Primack and Gallinat, 2016), i.e., episodes of unseasonably warm days in early spring followed by seasonally cold days (Gu et al., 2008; Man et al., 2009, 2013).

In summary, plant phenological responses to warming in spring are strongly influenced by temperatures at the time of spring events, i.e., larger responses are generally associated with lower temperatures and smaller responses with higher temperatures. Comparatively, early season species could have larger phenological changes but accumulate fewer forcing gains from these changes due to low temperatures. Total forcing gains added from higher temperatures would not differ among species when forcing gains between early and late season species are included. This suggests that phenological changes are not associated with changes in cumulative forcing temperatures and therefore would not be indicative of possible changes in plant growth, interspecific relationships, community structure, and ecosystem function that may occur with warming climate. The phenological assessments based on changes in timing of spring leaf-out and therefore active growing season would not provide adequate information about ecological implications of phenological changes. Future studies need to include temperatures at the time of spring events when reporting phenological changes.

Precautions may be required for possible limitations of the conclusions. First, our analyses were based on the assumption that plant phenology in spring is dependent only on temperatures, i.e., cumulative winter chilling for dormancy release and cumulative forcing temperatures for leaf-out, a hypothesis that most phenological models are based on (Murray et al., 1989; Harrington and Gould, 2015; Man et al., 2017, 2020; Zhang et al., 2018). There is increasing interest in the role of photoperiod on spring phenology (Körner and Basler, 2010; Polgar and Primack, 2011; Way and Montgomery, 2015). However, the effects of photoperiod are variable (Cooke et al., 2012; Wang et al., 2020) and certainly not as important as temperatures (Laube et al., 2014; Harrington and Gould, 2015; Nanninga et al., 2017). The claimed photoperiod effects are often confounded with temperature effects, i.e., longer photoperiods are associated with greater forcing temperatures (Nienstaedt, 1967; Worrall and Mergen, 1967; Falusi and Calamassi, 1990; Heide, 1993; Caffarra and Donnelly, 2011). More evident photoperiod effects are reported when winter chilling is insufficient (Körner and Basler, 2010; Way and Montgomery, 2015), which would not be the case with a moderate warming of 2°C in this study. In fact, cumulative winter chilling mostly increased (Table 2). Similarly, the effects of other environmental drivers, such as nutrient and water availability that are often limited to specific ecosystems, are generally smaller than those by temperatures or photoperiod (Piao et al., 2019). Second, we did not assess changes in leaf-fall time due to the lack of plant responses to warming in fall phenology (Piao et al., 2019) and the lack of a good understanding on among-species differences in leaf colouring/fall. We assumed that plant species would not differ in their responses to warming in fall phenology. However, there is evidence that spring and autumn phenology are positively correlated, i.e., plants that start early in spring will likely end early in fall producing little change in growing season length (Piao et al., 2019). This would support our conclusion that climate warming does not favour early season species.

## Data Availability Statement

The raw data supporting the conclusions of this article will be made available by the authors, without undue reservation.

## Author Contributions

RM and Q-LD conceived and designed the experiments. XC, RM, HZ, and WY collected the data. XC and RM wrote the code for modelling and projection. XC, RM, HZ, WY, JT, and Q-LD wrote the manuscript. All authors contributed to the article and approved the submitted version.

## Conflict of Interest

The authors declare that the research was conducted in the absence of any commercial or financial relationships that could be construed as a potential conflict of interest.

## Publisher’s Note

All claims expressed in this article are solely those of the authors and do not necessarily represent those of their affiliated organizations, or those of the publisher, the editors and the reviewers. Any product that may be evaluated in this article, or claim that may be made by its manufacturer, is not guaranteed or endorsed by the publisher.

## Footnotes

1
http://climate.weather.gc.ca/historical_data/search_historic_data_e.html

2
http://www.cma.gov.cn/2011qxfw/2011qsjgx/

## Supplementary Material

Supplementary Data Sheet 1 Study locations, species, and phenological models.

## References

[B1] Badeck F. W. Bondeau A. Böttcher K. Doktor D. Lucht W. Schaber J. (2004). Responses of spring phenology to climate change. *New Phytol.* 162 295–309.

[B2] Caffarra A. Donnelly A. (2011). The ecological significance of phenology in four different tree species: effects of light and temperature on bud burst. *Int. J. Biometeorol.* 55 711–721. 10.1007/s00484-010-0386-1 21113629

[B3] Cleland E. E. Allen J. M. Crimmins T. M. Dunne J. A. Pau S. Travers S. E. (2012). Phenological tracking enables positive species responses to climate change. *Ecology* 93 1765–1771. 10.1890/11-1912.1 22928404

[B4] Colombo S. J. (1998). Climatic warming and its effect on bud burst and risk of frost damage to white spruce in Canada. *For. Chron.* 74 567–577. 10.5558/tfc74567-4

[B5] Cooke J. E. K. Eriksson M. E. Junttila O. (2012). The dynamic nature of bud dormancy in trees: environmental control and molecular mechanisms. *Plant Cell Environ.* 35 1707–1728. 10.1111/j.1365-3040.2012.02552.x 22670814

[B6] Čufar K. De Luis M. Saz M. A. Črepinšek Z. Kajfež-Bogataj L. (2012). Temporal shifts in leaf phenology of beech (*Fagus sylvatica*) depend on elevation. *Trees* 26 1091–1100. 10.1007/s00468-012-0686-7

[B7] Falusi M. Calamassi R. (1990). Bud dormancy in beech (*Fagus sylvatica* L.). effect of chilling and photoperiod on dormancy release of beech seedlings. *Tree Physiol.* 6 429–438. 10.1093/treephys/6.4.429 14972934

[B8] Fitter A. H. Fitter R. S. R. (2002). Rapid changes in flowering time in British plants. *Science* 296 1689–1691. 10.1126/science.1071617 12040195

[B9] Gu L. Hanson P. J. Post W. M. Kaiser D. P. Yang B. Nemani R. (2008). The 2007 eastern US spring freeze: increased cold damage in a warming world? *Bioscience* 58 253–262. 10.1641/b580311

[B10] Gunderson C. A. Edwards N. T. Walker A. V. O’Hara K. H. Campion C. M. Hanson P. J. (2012). Forest phenology and a warmer climategrowing season extension in relation to climatic provenance. *Glob. Chang. Biol.* 18 2008–2025.

[B11] Hänninen H. (1990). Modelling bud dormancy release in trees from cool and temperate regions. *Acta For. Fenn.* 213 1–47. 10.1093/treephys/tpw061 27449791

[B12] Harrington C. A. Gould P. J. (2015). Tradeoffs between chilling and forcing in satisfying dormancy requirements for Pacific Northwest tree species. *Front. Plant Sci.* 6:120. 10.3389/fpls.2015.00120 25784922 PMC4347443

[B13] Heide O. M. (1993). Daylength and thermal time responses of budburst during dormancy release in some northern deciduous trees. *Physiol. Plant.* 88 531–540. 10.1111/j.1399-3054.1993.tb01368.x 28741760

[B14] Hollis D. McCarthy M. (2017). *UKCP09: Met Office Gridded and Regional Land Surface Climate Observation Datasets.* Available online at: http://catalogue.ceda.ac.uk/uuid/87f43af9d02e42f483351d79b3d6162a (accessed June 19, 2020).

[B15] Körner C. Basler D. (2010). Phenology under global warming. *Science* 327 1461–1462. 10.1126/science.1186473 20299580

[B16] Laube J. Sparks T. H. Estrella N. Höfler J. Ankerst D. P. Menzel A. (2014). Chilling outweighs photoperiod in preventing precocious spring development. *Glob. Chang. Biol.* 20 170–182. 10.1111/gcb.12360 24323535

[B17] Ma Q. Huang J. G. Hänninen H. Li X. Berninger F. (2021). Climate warming prolongs the time interval between leaf-out and flowering in temperate trees: effects of chilling, forcing and photoperiod. *J. Ecol.* 109 1319–1330.

[B18] Man R. Colombo S. Kayahara G. J. Duckett S. Velasquez R. Dang Q. L. (2013). A case of extensive conifer needle browning in northwestern Ontario in 2012: Winter drying or freezing damage? *For. Chron.* 89 675–680. 10.5558/tfc2013-120

[B19] Man R. Kayahara G. J. Dang Q. L. Rice J. A. (2009). A case of severe frost damage prior to budbreak in young conifers in Northeastern Ontario: consequence of climate change? *For. Chron.* 85 453–462. 10.5558/tfc85453-3

[B20] Man R. Lieffers V. J. (1997a). Seasonal photosynthetic responses to light and temperature in white spruce (*Picea glauca*) seedlings planted under an aspen (*Populus tremuloides*) canopy and in the open. *Tree Physiol.* 17 437–444. 10.1093/treephys/17.7.437 14759835

[B21] Man R. Lieffers V. J. (1997b). Seasonal variations of photosynthetic capacities of white spruce (*Picea glauca*) and jack pine (*Pinus banksiana*) saplings. *Can. J. Bot.* 75 1766–1771.

[B22] Man R. Lu P. (2010). Effects of thermal model and base temperature on estimates of thermal time to bud break in white spruce seedlings. *Can. J. For. Res.* 40 1815–1820. 10.1139/x10-129 33356898

[B23] Man R. Lu P. Dang Q. L. (2017). Insufficient chilling effects vary among boreal tree species and chilling duration. *Front. Plant Sci.* 8:1354. 10.3389/fpls.2017.01354 28861091 PMC5559465

[B24] Man R. Lu P. Dang Q. L. (2020). Effects of insufficient chilling on budburst and growth of six temperate forest tree species in Ontario. *New For.* 52 303–315. 10.1007/s11056-020-09795-1

[B25] Morin X. Lechowicz M. J. Augspurger C. O’Keefe J. Viner D. Chuine I. (2009). Leaf phenology in 22 American tree species during the 21st century. *Glob. Chang. Biol.* 15 961–975. 10.1111/j.1365-2486.2008.01735.x

[B26] Murray M. B. Cannell M. G. R. Smith R. (1989). Date of budburst of fifteen tree species in Britain following climatic warming. *J. Appl. Ecol.* 26 693–700. 10.2307/2404093

[B27] Nanninga C. Buyarski C. R. Pretorius A. M. Montgomery R. A. (2017). Increased exposure to chilling advances the time to budburst in North American tree species. *Tree Physiol.* 37 1727–1738. 10.1093/treephys/tpx136 29099953

[B28] Nienstaedt H. (1967). Chilling requirements in seven *Picea* species. *Silvae Genet.* 16 65–68.

[B29] Parmesan C. (2007). Influences of species, latitudes and methodologies on estimates of phenological response to global warming. *Glob. Chang. Biol.* 13 1860–1872. 10.1111/j.1365-2486.2007.01404.x

[B30] Peñuelas J. Rutishauser T. Filella I. (2009). Phenology feedbacks on climate change. *Science* 324 887–888. 19443770 10.1126/science.1173004

[B31] Piao S. Liu Q. Chen A. Janssens I. A. Fu Y. Dai J. (2019). Plant phenology and global climate change: current progresses and challenges. *Glob. Chang. Biol.* 25 1922–1940. 10.1111/gcb.14619 30884039

[B32] Polgar C. Gallinat A. Primack R. B. (2014). Drivers of leaf-out phenology and their implications for species invasions: insights from Thoreau’s concord. *New Phytol.* 202 106–115. 10.1111/nph.12647 24372373

[B33] Polgar C. A. Primack R. B. (2011). Leaf-out phenology of temperate woody plants: from trees to ecosystems. *New Phytol.* 191 926–941. 10.1111/j.1469-8137.2011.03803.x 21762163

[B34] Post E. Steinman B. A. Mann M. E. (2018). Acceleration of phenological advance and warming with latitude over the past century. *Sci. Rep.* 8:3927. 10.1038/s41598-018-22258-0 29500377 PMC5834618

[B35] Prevéy J. Vellend M. Rüger N. Hollister R. D. Bjorkman A. D. Myers-Smith I. H. (2017). Greater temperature sensitivity of plant phenology at colder sites: implications for convergence across northern latitudes. *Glob. Chang. Biol.* 23 2660–2671. 10.1111/gcb.13619 28079308

[B36] Primack R. B. Gallinat A. S. (2016). Spring budburst in a changing climate. *Am. Sci.* 104 102–109.

[B37] R Development Core Team (2019). *R Development Core Team. R version 3.6.1.*

[B38] Root T. L. Price J. T. Hall K. R. Schneider S. H. Rosenzweig C. Pounds J. A. (2003). Fingerprints of global warming on wild animals and plants. *Nature* 421 57–60. 10.1038/nature01333 12511952

[B39] Sarvas R. (1974). Investigations on the annual cycle of development of forest trees. II. autumn dormancy and winter dormancy. *Commun. Inst. For. Fenn.* 84 1–101.

[B40] Schwartz M. D. Ahas R. Aasa A. (2006). Onset of spring starting earlier across the Northern Hemisphere. *Glob. Chang. Biol.* 12 343–351. 10.1111/gcb.14001 29271095

[B41] Shen M. Tang Y. Chen J. Yang X. Wang C. Cui X. (2014). Earlier-season vegetation has greater temperature sensitivity of spring phenology in Northern hemisphere. *PLoS One* 9:e88178. 10.1371/journal.pone.0088178 24505418 PMC3914920

[B42] Suonan J. Classen A. T. Sanders N. J. He J. S. (2019). Plant phenological sensitivity to climate change on the Tibetan plateau and relative to other areas of the world. *Ecosphere* 10:e02543.

[B43] Tao J. Man R. Dang Q. L. (2021). Earlier and more variable spring phenology projected for eastern Canadian boreal and temperate forests with climate warming. *Trees For. People* 6:100127. 10.1016/j.tfp.2021.100127

[B44] Vitasse Y. Porté A. J. Kremer A. Michalet R. Delzon S. (2009). Responses of canopy duration to temperature changes in four temperate tree species: relative contributions of spring and autumn leaf phenology. *Oecologia* 161 187–198. 10.1007/s00442-009-1363-4 19449036

[B45] Vitasse Y. Signarbieux C. Fu Y. H. (2018). Global warming leads to more uniform spring phenology across elevations. *Proc. Natl. Acad. Sci. U.S.A.* 115 1004–1008. 10.1073/pnas.1717342115 29279381 PMC5798366

[B46] Wang H. Ge Q. Rutishauser T. Dai Y. Dai J. (2015). Parameterization of temperature sensitivity of spring phenology and its application in explaining diverse phenological responses to temperature change. *Sci. Rep.* 5:8833. 10.1038/srep08833 25743934 PMC4351518

[B47] Wang H. Wang H. Ge Q. Dai J. (2020). The interactive effects of chilling, photoperiod, and forcing temperature on flowering phenology of temperate woody plants. *Front. Plant Sci.* 11:443. 10.3389/fpls.2020.00443 32373144 PMC7176907

[B48] Way D. A. Montgomery R. A. (2015). Photoperiod constraints on tree phenology, performance and migration in a warming world. *Plant Cell Environ.* 38 1725–1736. 10.1111/pce.12431 25142260

[B49] Wolkovich E. M. Cook B. I. Allen J. M. Crimmins T. M. Betancourt J. L. Travers S. E. (2012). Warming experiments underpredict plant phenological responses to climate change. *Nature* 485 494–497. 22622576 10.1038/nature11014

[B50] Worrall J. Mergen F. (1967). Environmental and genetic control of dormancy in *Picea abies*. *Physiol. Plant.* 20 733–745.

[B51] Zettlemoyer M. A. Schultheis E. H. Lau J. A. (2019). Phenology in a warming world: differences between native and non-native plant species. *Ecol. Lett.* 22 1253–1263. 10.1111/ele.13290 31134712

[B52] Zhang H. Liu S. Regnier P. Yuan W. (2018). New insights on plant phenological response to temperature revealed from long-term widespread observations in China. *Glob. Chang. Biol.* 24 2066–2078. 10.1111/gcb.14002 29197142

[B53] Zhang H. Yuan W. Liu S. Dong W. Fu Y. (2015). Sensitivity of flowering phenology to changing temperature in China. *J. Geophys. Res. Biogeosci.* 120 1658–1665.

[B54] Ziello C. Estrella N. Kostova M. Koch E. Menzel A. (2009). Influence of altitude on phenology of selected plant species in the alpine region (1971–2000). *Clim. Res.* 39 227–234.

