# Supplementary material for: Does Climate Warming Favour Early Season Species?
Source: Front Plant Sci. 2021 Nov 18;12:765351. doi: 10.3389/fpls.2021.765351 (PMC8639222; doi:10.3389/fpls.2021.765351)
Supplement: Supplementary Data Sheet 1 — Study locations, species, and phenological models. [file Data_Sheet_1.pdf]

Supplementary materials

Table S1. Climate regions and locations used to compare the responses of plant species with differing spring phenology to climate warming.

**Climate region/location**	**Latitude and longitude**	**Annual mean temperature (°C) (1981 – 2010)**
	**Boreal Canada**	
St. John's, Baie-Comeau, Timmins, Kapuskasing, Sioux Lookout, Thompson, Fort McMurray, La Ronge, High level, Fort Nelson, Yellowknife, Whitehorse	47°37′ – 62°28′N; 52°44′ – 135°04′W	-4.3 – 5.0
	**Temperate Canada**	
London, Toronto, Montreal, Sherbrooke, Quebec City Ottawa, Sudbury, North Bay, Sault Ste. Marie	43°01′ – 46°48′N; 71°23′ – 84°30′W	4.0 – 7.9
	**Cold temperate China**	
Shenyang, Changchun, Tonghua, Baicheng, Xilingol, Shihezi, Jiamusi, Mudanjiang, Harbin, Yichun, Dedu, Nenjiang	41°25′ – 49°06′N; 85°56′ – 130°11′E	0.1 – 8.5
	**Warm temperate China**	
Baoji, Luoyang, Taian, Jinan, Xingtai, Dezhou, Minqin, Taiyuan, Tianjin, Yixian, Qinhuangdao, Beijing	34°13′ – 39°31′N; 103°03′ – 119°37′E	8.8 – 14.8
	**Temperate United Kingdom**	
Belfast, Harris, Thurso, Inverness, Aberdeen, Edinburgh, Sunderland, Chester, Norwich, Brighton, Plymouth, Pembroke	50°49'– 58°35'N; 0°08' – 7°18W	7.8 – 11.2

Table S2. Phenological models for different species/specie groups listed by increasing forcing requirements (early to late season species) within each climate region.

Species	Model[a]	Parameters (a, b, c, d)
	**Boreal Canada**	
Pinus banksiana	3-exponential	2013, 10993, 0.0049
Populus balsamifera	4-sigmoid	3266, 51776, 247, -107
Betula papyrifera	3-exponential	5819, 50394, 0.0054
Populus tremuloides	4-sigmoid	6217, 53076, 304, -115
Picea glauca	3-exponential	6505, 21558, 0.0080
Picea mariana	3-exponential	8768, 18145, 0.0118
	**Temperate Canada**	
Pinus strobus	4-sigmoid	1438, 29274, 429, -165
Larix laricina	4-sigmoid	2769, 199142, -297, -248
Betula alleghaniensis	4-sigmoid	1311, 88151, 332, -263
Pinus resinosa	4-sigmoid	5175, 113800, 17, -221
Thuja occidentalis	4-sigmoid	5742, 80365, 376, -124
Picea rubens	4-sigmoid	10771, 59227, 430, -176
	**Cold temperate China**	
Salix babylonica	2-exponential	104.2, 0.0170
Salix matsudana	2-exponential	117.4, 0.0266
Populus simonii	2-exponential	127.8, 0.0139
Ulmus pumila	2-exponential	86.5, 0.0080
Populus X canadensis	2-exponential	375.1, 0.0090
Amorpha fruticosa	2-exponential	294.6, 0.0078
Koelreuteria paniculata	2-exponential	175.7, 0.0110
Fraxinus chinensis	2-exponential	136.1, 0.0048
Ailanthus altissima	2-exponential	210.1, 0.0088
Ginkgo biloba	2-exponential	256.8, 0.0156
Morus alba	2-exponential	284.1, 0.0110
Sophora japonica	2-exponential	115.6, 0.0083
	**Warm temperate China**	
Prunus davidiana	2-exponential	190.4, 0.0178
Platycladus orientalis	2-exponential	385.6, 0.0150
Juglans regia	2-exponential	263.7, 0.0183
Malus pumila	2-exponential	305.1, 0.0071
Euonymus alatus	2-exponential	61.2, 0.0457
Prunus kansuensis	2-exponential	182.5, 0.0107
Amygdalus persica	2-exponential	161.0, 0.0110
Robinia pseudoacacia	2-exponential	162.1, 0.0065
Gleditsia sinensis	2-exponential	177.1, 0.0099
Toona sinensis	2-exponential	221.1, 0.0083
Ziziphus jujuba	2-exponential	174.7, 0.0075

Pinus tabuliformis	2-exponential	724.2, 0.0094
**Temperate United Kingdom**		
Group 1[b]	3-exponential	41.4, 548, 0.0391
Group 2	3-exponential	35.3, 410, 0.0228
Group 3	3-exponential	34.8, 545, 0.0200
Group 4	3-exponential	19.1, 664, 0.0142
Group 5	3-exponential	-15.1, 1143, 0.0139

[a]Three model types, 2-parameter exponential decay, y=a*exp(-bx) (Zhang et al., 2018), 3-parameter exponential decay, y=a + b*exp(-cx) (Murray et al., 1989; Man et al., 2017), and 4-parameter sigmoid curve, $y=a + b*(1+ \exp((x-c)/d)^{-1}$ (Man et al., 2020).

[b] Group 1 – *Populus trichocarpa* and *Crataegus monogyna*; Group 2 – *Sambucus nigra*, *Rosa rugosa*, *Salix viminalis*, *Larix decidua*, and *Prunus avium*; Group 3 – *Rubus idaeus*, *Sorbus aucuparia*, *Betula pendula*, and *Corylus avellane*; Group 4 – *Robina pseudoacacia*, *Tsuga heterophylla*, and *Picea sitchensis*; and Group 5 – *Fagus sylvatica*. As the reported model parameters are not consistent with the model types presented, models were refitted with data extracted from the graphical presentations using the distance measuring tool in Adobe Acrobat Reader DC.
